# CPM-Related Mechanisms Could Play a Key Role in the Effects on Pain Sensitivity Induced by Manual Therapy: Three Crossover Trials Investigating the Effects of Manual Pressure

**DOI:** 10.3390/jcm13133648

**Published:** 2024-06-22

**Authors:** Alberto Arribas-Romano, Josué Fernández-Carnero, Leonardo Rodríguez-Lagos, Miguel Molina-Álvarez, Jesús Zabala-Zambrano, Lucas Lezaun-Hernández, Lucía Contreras-Padilla, Francisco Mercado

**Affiliations:** 1 Escuela Internacional de Doctorado, Department of Physical Therapy, Occupational Therapy, Rehabilitation and Physical Medicine, Universidad Rey Juan Carlos, 28933 Madrid, Spain; alberto.arribas@urjc.es (A.A.-R.); l.rodriguezla.2019@alumnos.urjc.es (L.R.-L.); 2 Cognitive Neuroscience, Pain and Rehabilitation Research Group (NECODOR), Faculty of Health Sciences, Rey Juan Carlos University, 28922 Madrid, Spain; miguel.molina@urjc.es (M.M.-Á.); francisco.mercado@urjc.es (F.M.); 3 Department of Physical Therapy, Occupational Therapy, Rehabilitation and Physical Medicine, Rey Juan Carlos University, 28922 Madrid, Spain; 4 Motion in Brains Research Group, Institute of Neuroscience and Movement Sciences (INCIMOV), Centro Superior de Estudios Universitarios La Salle, Universidad Autonoma de Madrid, 28049 Madrid, Spain; 201011139@campuslasalle.es (J.Z.-Z.); 201011074@campuslasalle.es (L.L.-H.); 5 La Paz Hospital Institute for Health Research, IdiPAZ, 28029 Madrid, Spain; 6 Area of Pharmacology, Nutrition and Bromatology, Department of Basic Health Sciences, Rey Juan Carlos University, Unidad Asociada I+D+i Instituto de Química Médica (IQM) CSIC-URJC, 28922 Alcorcón, Spain; 7 Advance Rehabilitation Center Sanitas, 28046 Madrid, Spain; 8 Edurne Esquide Fisioterapia, 31200 Estella, Spain; 9 iCentro Fix You, 28009 Madrid, Spain; lucia.contrerasp@estudiante.uam.es; 10 School of Physiotherapy ONCE, Universidad Autónoma de Madrid, 28022 Madrid, Spain; 11 Department of Psychology, Faculty of Health Sciences, Universidad Rey Juan Carlos, 28922 Madrid, Spain

**Keywords:** manual therapy, conditioned pain modulation, pain mechanisms

## Abstract

**Objective**: The aim of this study is to assess whether pain-inducing manual pressure (PIMP) leads to effects on pressure pain threshold (PPT) mediated by conditioned pain modulation (CPM) and whether these effects are influenced by the intensity and repetition of the stimulus. Additionally, the influence of psychological factors and physical activity on the response to PIMP was explored. **Methods**: A total of 72 pain-free students were randomly assigned to three crossover trials. Trial 1 compared the effects of PIMP with the cold pressor task and pain-inducing electrostimulation. Trial 2 compared the effects of manual pressure that elicited moderate pain, mild pain, and no pain. Trial 3 compared a single PIMP stimulation with four stimuli applied at the same site or at different sites. **Results**: PIMP produced a lower increase in PPT than cold pressor task and no difference with electrostimulation. Manual pressure that caused moderate pain led to a greater increase in PPT compared to mild pain and pain-free application. Repetition of PIMP stimulus, whether at the same or different sites, did not significantly increase PPT compared to a single stimulation. No association with psychological factors or physical activity was found. **Conclusions**: PIMP produces an increase in PPT, suggesting the involvement of CPM-related mechanisms.

## 1. Introduction

Chronic pain is a disease with a high socio-health impact that increases with age and is more prevalent among women [1,2]. Manual therapy (MT) is a non-pharmacological intervention preferred and most used by physiotherapists for the management of the most prevalent musculoskeletal pain conditions such as low back and neck pain [3]. Although the mechanisms involved in MT are largely unknown, its short-term effects and safety profile make it an attractive therapeutic tool with a moderate recommendation grade in some clinical practice guidelines [4,5,6]. However, studies show very heterogeneous results regarding its efficacy in the pain treatment [7,8], and it has been suggested that this is due to a substantial inter-individual variability in response to MT [9]. A possible explanation for this variability is the different responses of endogenous pain mechanisms to treatment [9,10,11]. Therefore, a mechanism-based approach has been proposed to help find patients that can benefit from MT and allow for a better personalized treatment. However, this first requires an understanding of the mechanisms involved in the effects of MT, which remain unknown [9].

It has been proposed that the mechanical stimulus of MT initiates a series of potential neurophysiological effects causing a pain inhibitory response [9,12]. Meta-analyses using quantitative sensory testing (QST) have reported significant immediate effects of MT on both static and dynamic pain modalities in individuals with musculoskeletal pain [13,14]. This modulation of pain sensitivity of MT has been associated with beneficial effects on clinical pain, which may partly explain the improvement in patients’ symptoms [15]. Conditioned pain modulation (CPM), a paradigm exploring the phenomenon of “pain inhibits pain” in humans [16], involves the application of a painful conditioning stimulus (COS) to inhibit or decrease the intensity of evoked pain or increase the pain threshold to another noxious stimulus applied in a different body area (test stimulus) [16,17]. This paradigm represents an approach to diffuse noxious inhibitory controls (DNIC), an endogenous pain modulation mechanism observed in animal models. However, it is important to note that although related, the paradigm is not equivalent to CPM [18]. Pain-inducing manual pressure (PIMP) applied in certain MT techniques may activate CPM-related mechanisms, potentially explaining the immediate effects on pain sensitivity. This hypothesis was explored by Wilson et al. (2021) [19] in healthy participants by comparing the effects on pain sensitivity between PIMP, CPM paradigm assessed with cold pressor task (CPT) and pain-free manual pressure. The findings revealed that PIMP produced an effect similar to the CPM paradigm in foot PPT and significantly greater than those of pain-free manual pressure. However, they did not find any group-by-time interaction in terms of changes in mechanical and thermal sensitivity in the forearm and upper back.

Factors such as intensity, duration and dose of pain induced by a COS could have a significant influence on the inhibitory response. However, mixed results have been found regarding the influence of intensity [20,21,22,23,24,25], as well as a non-significant impact of duration [25,26]. To our knowledge, no study has evaluated the influence of the intensity and dose of PIMP stimulus on pain sensitivity. 

Additionally, individual factors may also influence the inhibitory response to PIMP. Age and gender have been shown to be associated with the efficacy of CPM, but the influence of other factors such as psychological state or level of physical activity is unclear [27,28]. In the CPM paradigm, the subjective experience of pain plays a key role and could therefore be influenced by anxiety, catastrophizing or fear of pain. The rationale for investigating the influence of physical activity is that regular exercise has been shown to increase concentrations of neurotransmitters involved in pain inhibition and facilitation processes, such as endogenous opioids [29,30,31].

In the present study, three crossover clinical trials were performed with the aim of investigating the effects of PIMP on pain sensitivity. The aim of Trial 1 was to compare the immediate changes in PPT of PIMP with the CPM paradigm evaluated with the cold pressure task (CPT) and with another pain-inducing therapeutic stimulus, like electrical stimulation. The aim of Trial 2 was to investigate the influence of the intensity of manual pressure on PPT, comparing pressure that induces moderate pain, pressure that induces mild pain and pressure that does not induce pain. Trial 3 aimed to investigate the influence of repetition of PIMP stimuli on PPT, comparing a single stimulus with four stimuli at the same point and with four stimuli at different points. Finally, the influence of state anxiety, pain anxiety, fear of pain, pain catastrophism and physical activity level on changes in PPT produced by PIMP was explored.

## 2. Materials and Methods

### 2.1. Study Design

Three separate randomized crossover clinical trials were conducted in accordance with the guidelines outlined in the Consolidated Standards of Reporting Trials (CONSORT) statement [32]. The trials took place at three institutions: Rey Juan Carlos University, LaSalle University Centre, and the ONCE University School of Physiotherapy. The procedures followed were in accordance with the Ethics Committee of Rey Juan Carlos University (protocol code: 1901202203622 and date of approval: 14 March 2022) and with the Helsinki Declaration (1964, amended most recently in 2013) of the World Medical Association. To ensure transparency and accessibility of the trial, they have been registered with ClinicalTrials.gov under the respective identifiers NCT05730127, NCT05730166, and NCT05730179.

### 2.2. Participants

Participants were recruited via emails, posters on social networks and oral presentations in university classrooms. Included were healthy participants aged 18–65 years who were free of pain and had no history of chronic pain. Exclusion criteria were pregnancy or having given birth within the last year.

### 2.3. Outcome Measures

#### 2.3.1. Baseline Characteristics

Sex, age, weight and height were collected at baseline. In addition, participants completed the following questionnaires: the Pain Catastrophizing Scale (PCS), to assess catastrophizing cognitions and behaviors concerning pain [33]; the State Anxiety Inventory (STAI-S), to measure of anxiety as a state [34,35,36]; the Pain Anxiety Symptoms Scale (PASS-20), to assess anxiety about pain [37,38]; the Fear of Pain Questionnaire-III (FPQ-III) to assess the fear of pain [39,40]; and the International Physical Activity Questionnaire (IPAQ), to assess levels of physical activity [41].

#### 2.3.2. Pressure Pain Thresholds

PPTs were assessed on the tibialis anterior muscle of the dominant side using a digital algometer (Model FPX, Wagner instruments, Greenwich, CT, USA). Participants were instructed to say “stop” when the pressure sensation became painful. Three assessments were performed on the tibialis muscle on the dominant side with a washout time of 30 s in between. The pressure exerted by the algometer was gradually increased at a rate of 1 kg/s. Data were collected in kg/cm^2^, and the average of the three assessments was calculated for further analysis.

### 2.4. Procedure

Participants who met the inclusion criteria were sent the information sheet and had to sign an informed consent form before partaking in the study. Participants were randomly assigned to one of the three clinical trials and to one of the three intervention sequences with a 1:1:1:1:1:1:1:1:1 allocation ratio. One of the interventionists generated a table with a random number of 0–9 (3 groups × 3 sequences) assigned to each participant ID code with the quick calculation tool of www.graphpad.com (accessed on 10 February 2023). The assignment of each ID code was hidden separately in a folder accessible only to the interventionists. The recruitment and consecutive assignment of participant ID codes was performed by blinded investigators who would later conduct the assessment. The interventionists were unaware of each participant’s ID code until the time of the first intervention, in order to conceal the assignment from them until that time.

**Figure 1:**
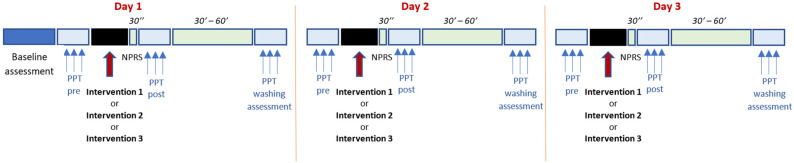
Experimental session timeline.

Participants attended three days in a week, one for each intervention, allowing at least 1 day between sessions as a washout period. On day 1, they first completed demographic data and questionnaires. The following assessment and treatment protocol was then performed and was the same on all three days (Figure 1).

First, a blinded assessor carried out the three tibial PPT assessments with a washout period of 30 s; subsequently, the assessor left the room. Then, a physiotherapist specializing in manual therapy performed the intervention assigned to the participant for that day. Immediately after the procedure, the participant was asked to rate the average pain intensity experienced during the intervention using a numerical pain rating scale (NPRS), ranging from 0 “no pain to 10 “worst imaginable pain”. After a few seconds, the assessor entered the room and reassessed the PPT three times 30 s after the intervention. Finally, the patient was asked to return 30 to 60 min later, and the PPT was re-evaluated to see whether there was a washout effect after this time.

### 2.5. Interventions Trial 1

*PIMP.* The physiotherapist placed his thumb on the midpoint of the non-dominant trapezius muscle between the acromion and C7 and applied progressive pressure until the participant reported a pain intensity of 5/10 on the NPRS scale. The participant was instructed to give continuous feedback of the perceived pain intensity, and if they forgot, the physiotherapist asked every 30 s. The physiotherapist adapted the intensity of the pressure to try to maintain the 5/10 pain. This was the experimental intervention across all 3 trials.

*Pain-inducing electrical stimulation.* Electrical stimulation was applied with a TENS device (Premier TENS plus, Med Fit) at a frequency of 100 Hz and a bandwidth of 150 μs. The patient was instructed to adapt the intensity to perceive 5/10 pain intensity in the NPRS for two minutes. The electrodes were positioned on either side of the midpoint of the non-dominant trapezius muscle.

*CPT.* The participant placed the non-dominant hand in a bath of cold water for two minutes. The water temperature was set to 10.5 ± 0.5 °C to try to achieve a pain intensity of 5/10 in the NPRS, based on previous studies [42] and tests.

### 2.6. Interventions Trial 2

PIMP, which elicited a moderate pain level rated at 5/10 on the numerical pain rating scale (NPRS), was compared to manual pressure rated at 2/10 and to manual pressure that did not elicit any pain, rated at 0/10. The patient’s continuous feedback was assessed as previously described.

### 2.7. Interventions Trial 3

A single stimulus of 2 min of manual pressure intervention on the trapezius at an intensity that elicited a rated pain intensity of 5/10 for two minutes was compared with the following:

*Repeated PIMP at the same point.* Two minutes of manual pressure at 5/10 on the trapezius were repeated four times with one minute rest between stimuli.

*Repeated PIMP at different points.* The first manual pressure was applied to the non-dominant upper trapezius, the second to the dominant trapezius, the third to the non-dominant iliocostalis lumborum and the fourth to the dominant iliocostalis lumborum. Each stimulus lasted for a duration of two minutes, with one minute of rest provided between stimuli. 

The patient’s continuous feedback was evaluated using the previously described method.

### 2.8. Statistical Analysis

Baseline characteristics of each trial were summarized with descriptive statistics such as mean, standard deviation, median and interquartile ranges. To assess differences in baseline characteristics between samples for each trial, an analysis of variance (ANOVA) was used for normally distributed continuous variables. The Kruskal–Wallis test was performed for non-normally distributed continuous variables and the Chi^2^ test for the categorical variable sex.

To assess differences between interventions within each trial, a mixed-effects model analysis was used. Post-intervention PPT was entered as the dependent variable and the intervention as the independent variable, adjusted for the following covariates: day of intervention, sequence, and pre-intervention PPT. Day and sequence were introduced into the model to control for possible carryover effect, period effect, sequence effect, and period-by-treatment interaction. All subjects were considered in the analysis even if they had missing data on some of the days, as the mixed-effects model has proven to be a valuable tool for the analysis of crossover trials with incomplete data [43]. In addition, a sensitivity analysis was performed only on participants without missing data to check whether the same conclusions were reached. Bonferroni-adjusted post hoc contrasts were carried out to assess differences between the interventions, days, and sequences. Significance was set at *p* < 0.05. In Trial 1, due to the unexpected significant differences in participants’ perceived pain intensity across the different types of intervention, an analysis adjusting for this was conducted to explore its possible influence.

The restoration of the PPT minutes after the interventions was explored by analyzing the differences between the baseline PPT and the PPT between 30 and 60 min after the intervention with a paired Student’s *t*-test. Significance was set at *p* < 0.01 due to the multiple comparisons made. In addition, given the possibility of a progressive sensitization of the measurement point, the efficacy of the washout period was evaluated by analyzing the differences between the baseline PPT on each of the days with an ANOVA test. Bonferroni-adjusted post hoc contrasts were carried out, and significance was set at *p* < 0.05.

The associations between psychosocial factors and the PIMP effect in PPT were explored using a multiple regression model. This included subjects from the three trials who had no missing data on the day of the intervention. Post-intervention PPT was entered as dependent variable, psychosocial factor as an independent variable, and pre-intervention PPT, sex and age as covariates. Sex and age were controlled for because their influence on CPM magnitude has been suggested [27]. The same model was used to assess the possible association with weekly physical activity. Significance was set at *p* < 0.05.

### 2.9. Sample Size Calculation

The sample size was calculated to detect a greater than 30% change in the tibialis anterior (1.8 kg/cm^2^) [44] and greater than minimum detectable change [45], with a standard deviation of 1.8 based on a previous study on a similar population [44]. With a significance level of 0.05 and 80% power, 21 participants were required in each group/trial. Estimating a loss ratio of 10%, 24 students per group/trial were finally recruited. 

Statistical analyses including sample size calculation were conducted in STATA (IC 16.1, StataCorp LLC, Lakeway Drive College Station, TX, USA).

## 3. Results

**Figure 2:**
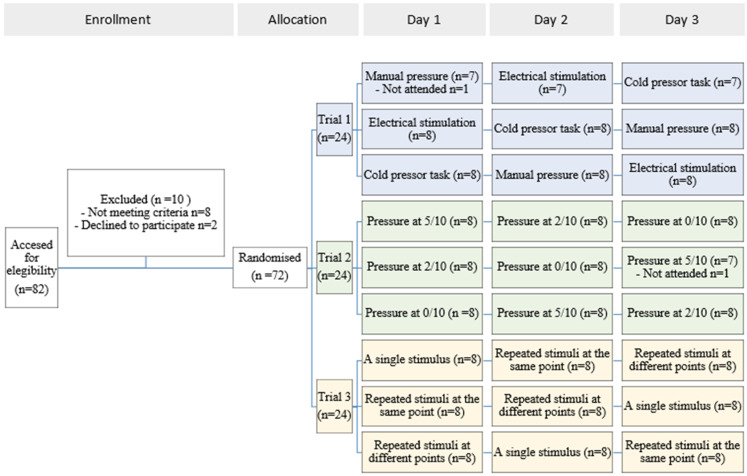
Participant flow diagram.

A total of 72 healthy volunteers participated in this project; 24 participants were randomly assigned to each trial, 8 to each sequence of interventions. There was one dropout in Trial 1 and one in Trial 2 (see participant flow chart) (Figure 2). 

There were no significant differences in the baseline characteristics of participants between trials (Table 1).

### 3.1. Trial 1

A total of 24 students participated in Trial 1, and there was only 1 dropout. Trial 1 therefore had 23 participants: 7 students in sequence 1, 8 students in sequence 2 and 8 students in sequence 3. All 23 participants completed all three days (Figure 2). Out of the 23 participants, 4 subjects had missing data on their pre- or post-intervention PPT on at least one of the days.

Statistically significant differences (*p* = 0.008) were found in mean pain intensity perceived by participants between PIMP (mean = 4.91; SD = 0.67), pain-inducing electrical stimulation (mean = 4.85; SD = 0.73), and CPT (mean = 5.95; SD = 2.01).

**Figure 3:**
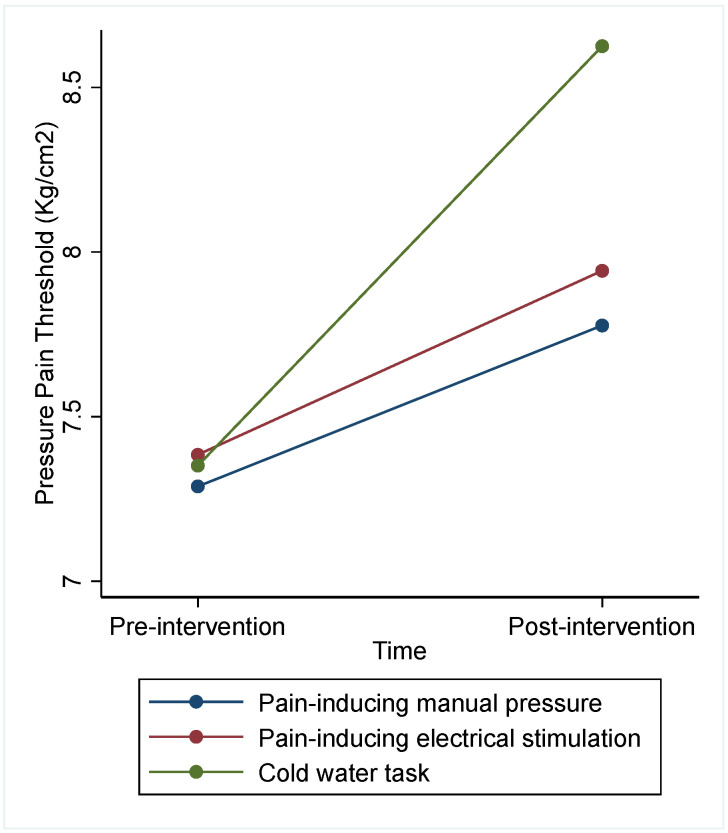
PPT before and after each of the interventions in Trial 1.

The analysis considering all subjects showed a significant effect for type of intervention (Chi^2^ = 7.76; *p* = 0.021), but not for day (Chi^2^ = 2.53; *p* = 0.283) or sequence (Chi^2^ = 0.38; *p* = 0.827) (Table 2 and Figure 3). CPT showed a significantly greater increase in PPT than the PIMP (difference = −0.77; CI 95% [−1.53 to −0.02]). Analysis adjusted for participants’ perceived pain intensity did not show a significant influence of pain intensity (coef. = 0.01; *p* = 0.972). However, the analysis only considering subjects with no missing data showed no significant effect on the intervention factor (Chi^2^ = 5.92; *p* = 0.052), nor did it find significant differences between CPT and PIMP (difference = −0.72; CI 95% [−1.53 to 0.09]) (see Table S1 and Figure S1 in the Supplementary Materials).

### 3.2. Trial 2

A total of 24 students participated in Trial 2, and 1 did not attend the third day. Trial 2 therefore had 23 participants: 8 students from sequence 1, 7 students from sequence 2 and 8 students from sequence 3. All 23 participants completed all three days (Figure 2). Out of the 23 participants, 2 subjects had missing data in their pre- or post-intervention PPT on at least one of the days.

The mean (SD) intensity of pain perceived by participants was 4.66 (1.08) from manual pressure aiming to cause 5/10 pain, 1.93 (0.23) from manual pressure aiming to cause 2/10 pain and 0.02 (0.10) from manual pressure aiming not to cause pain.

**Figure 4:**
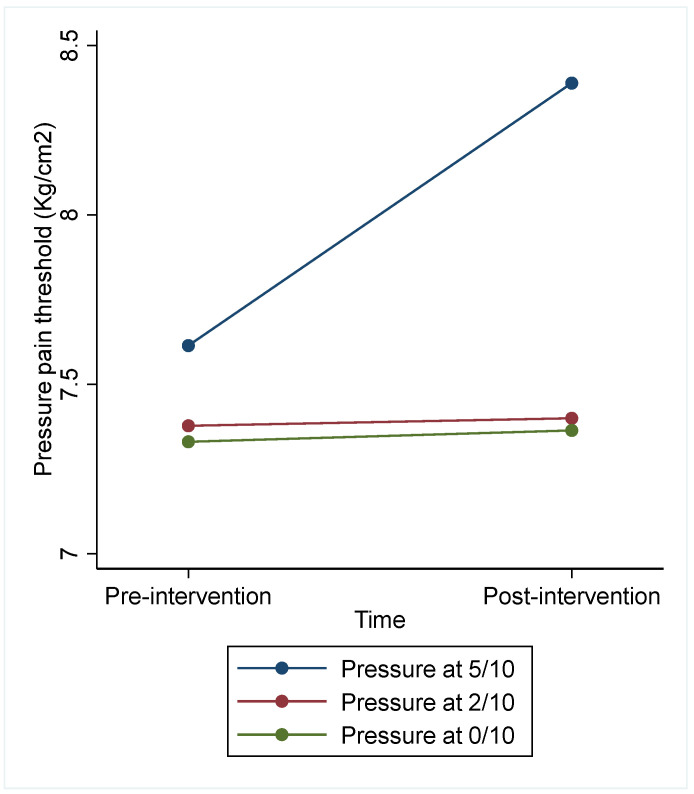
PPT before and after each of the interventions in Trial 2.

The analysis considering all subjects showed a significant effect for type of intervention (Chi^2^ = 9.72, *p* = 0.008). However, we found no effects regarding the day (Chi^2^ = 2.02, *p* = 0.364), or the sequence (Chi^2^ = 1.10, *p* = 0.577) (Table 3 and Figure 4). Manual pressure at 5/10 showed a significant increase in PPT in comparison to manual pressure at 2/10 (difference = 0.79; 95% CI [0.09 to 1.48]) and pain-free manual pressure (difference = 0.79; 95% CI [0.09 to 1.49]). Sensitivity analysis considering only subjects with no missing data led to similar conclusions (see Table S2 and Figure S2 in the Supplementary Materials).

### 3.3. Trial 3

A total of 24 students participated in Trial 3 and all 24 participants attended all three days (Figure 2). Two subjects had missing data on the pre- or post-intervention PPT on at least one of the days.

The mean (SD) intensity of pain perceived by participants for single-point manual pressure 1 × 2 min was 4.96 (0.79), for repeated manual pressure at the same point 4 × 2 min was 4.81 (0.70), and for repeated manual pressure at different points 4 × 2 min was 4.77 (0.78).

**Figure 5:**
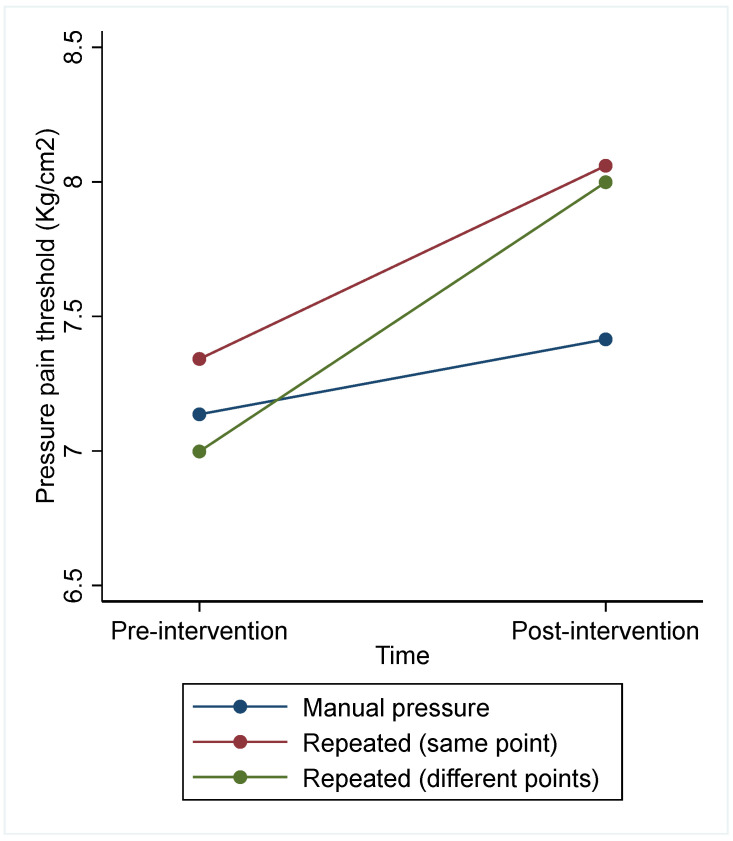
PPT before and after each of the interventions in Trial 3.

The analysis of the three interventions considering all subjects found no significant effects of intervention factor (Chi^2^ = 5.51, *p* = 0.064), day (Chi^2^ = 0.48, *p* = 0.787), or sequence (Chi^2^ = 0.97, *p* = 0.616) (Table 4 and Figure 5). Manual pressure 1 × 2 min showed no difference with either repeated manual pressure at the same point (difference = −0.45, 95% CI [−1.19 to 0.28]) or with repeated manual pressure at different points (difference = −0.72, 95% CI [−1.46 to 0.02]). Sensitivity analysis considering only subjects with no missing data led to similar conclusions (see Table S3 and Figure S3 in the Supplementary Materials).

### 3.4. PPT Washout Period

No differences were found between baseline PPT and PPT 30–60 min after any of the interventions. In addition, no differences were also found between the baseline PPT of the 3 intervention days (see Tables S4 and S5 of the Supplementary Materials).

### 3.5. Associations between Psychological Factors or Physical Activity Level and the Effect on PPT Produced by PIMP

Psychological factors and physical activity were not significantly associated with the effects of PIMP on PPT (beta values between −0.01 and 0.03: *p* ≥ 0.310) (Table 5).

## 4. Discussion

The aim of this trial was to investigate the effect on pain sensitivity of a PIMP stimulus commonly applied in manual therapy treatments. The results suggest that PIMP may induce a smaller increase in PPT than the CPM paradigm assessed with CPT but a similar PPT to pain-inducing electrostimulation. Manual pressure induced at moderate pain produced a significantly greater change in PPT compared to that induced at mild pain or pain-free.. Performing more repetitions (of manual pressure-induced pain), either at the same body point or at different points, did not result in a significantly greater increase in PPT, in comparison to performing it only once. Finally, the effect of PIMP on PPT was not associated with anxiety status, pain anxiety, fear of pain, catastrophizing, or physical activity level.

The results of Trial 1 suggest that PIMP may induce a smaller inhibitory effect than that produced by CPT (commonly used to evaluate the CPM paradigm). Despite this, in Trial 2, manual pressure inducing moderate pain generated a significantly greater increase in PPT than pain-free manual pressure. Trial 2’s findings support the hypothesis that these effects on pain sensitivity may be mediated by CPM-related mechanisms. Wilson et al. [19] reached a similar conclusion with PPT in the foot of healthy participants; however, their findings showed a similar effect in PIMP and CPT. Therefore, Trial 2’sbatr results demonstrate that the immediate and short-term effects of MT could be partly explained by endogenous inhibitory processes initiated after the application of techniques involving painful manual pressure. Although Trial 2 showed promising results, it is important to note that both Wilson et al. [19] (2021)’s study and Trial 2 were conducted in healthy people who have been shown to have a more effective CPM response than patients with chronic pain [46,47]. In a previous study in which pain-free MT was compared to painful MT, this hypothesis was evaluated but in subjects with chronic or recurrent neck pain. Painful MT did not produce an immediate increase in PPTs, nor differences in effect compared to pain-free MT [48]. Not all patients with chronic pain appear to have alterations in pain processing, and it is suggested that responses to treatment mediated by these endogenous pain mechanisms may vary between patients [11,49]. Therefore, future studies should investigate which patient phenotypes may benefit from these immediate effects on pain sensitivity related to PIMP.

Previous studies have also reported that the type of COS has an influence on pain sensitivity [50,51]. Oono et al. (2011) found that CPT induced a higher CPM magnitude than the pressure cuff and mechanical head pressure. Their results were similar to those of Trial 1, and they also found that CPT induced greater pain. This increase in pain could potentially explain the larger effect observed. However, in a secondary analysis, the influence of this induced pain on the results was found to be negligible. Similar results were reported by Aparecida da Silva, Galhardoni, Teixeira, and Ciampi de Andrade (2018) who, controlling for induced pain intensity, showed that the CPT induced a higher CPM magnitude than the ischemic cuff. These findings suggest that cold water pain stimulation elicits a greater increase in PPT compared to mechanical or ischemic stimuli. Thus, the magnitude of the inhibitory response triggered by painful stimulation may be influenced by the specific type of stimulus applied.

The results obtained from Trial 2 suggested that the intensity induced with manual pressure is relevant. This trial found a significantly greater effect when inducing 5/10 pain compared to 2/10 pain. These differences between stimulation at moderate and mild pain intensity have also been reported using noxious stimuli elicited by an ischemic cuff [52] and contact heat [20]. However, studies have yielded conflicting results regarding the relationship between the magnitude of conditioned pain modulation (CPM) and the intensity of pain induced by the COS. Some studies have reported an association [23,24,25], while others have not found a significant association [20,21,22]. It is also unclear what minimum pain intensity is necessary to activate these mechanisms in healthy people. The results from Trial 2 suggest that manual pressure at 2/10 is not sufficient to elicit the inhibitory mechanisms involved in CPM. This supports expert guidelines that recommend the COS should elicit a pain intensity greater than 2/10 in order to generate a CPM response [16]. It is possible that the magnitude of response depends on the noxiousness of the COS and not on the intensity of pain induced, as it has been found that a strong but subjectively non-painful stimulation can trigger pain-inhibiting effects [53]. Some studies have reported an association between the physical intensity of the COS and the inhibitory response. These results have been observed in healthy volunteers assessing the CPM response [54] and in animals assessing the DNIC response [55]. These intensity-related hypoalgesic effects in healthy participants have also been found with exercise, where higher intensity results in greater hypoalgesia [56,57], but it is also unknown which specific parameters would be the most optimal. In pain populations, joint mobilizations have shown mixed effects in terms of the influence of intensity on the effect on PPTs [58,59]. Among patients with knee osteoarthritis, mobilization techniques that involved more intense movement toward the end of the range of motion resulted in a significantly greater change in PPTs compared to techniques that did not reach the end of the range [58]. Nevertheless, in patients with neck pain, there was no difference in PPT when performing high-force or low-force mobilization [59].

The findings from Trial 3 did not show any influence in the effect of increasing the dose on PPT. This is consistent with findings from previous studies in which the duration of COS did not influence the magnitude of CPM assessed with PPTs [25,26]. Razavi, Hansson, Johansson, and Leffler (2014) found that CPM responses were unaffected by different durations of ischemic cuff- or contact heat-induced pain (between 0 to 6 and 6 to 12 min). Smith and Pedler (2018) found no significant effect of the duration for which the ischemic cuff was maintained inflated on the CPM magnitude. In addition, Trial 3 also found no significantly greater effect of repeated stimulation at different points on the body. These findings suggest that the possible effect on pain sensitivity of manual pressure related to mechanisms involved in CPM may have a ceiling effect within a few minutes, regardless of the area in which pain is induced.

Regression analyses showed no significant association for any of the psychological factors analyzed. This is consistent with meta-analyses conducted by Nahman-Averbuch, Nir, Sprecher, and Yarnitsky (2016), who found no association between CPM magnitude and state anxiety or catastrophizing in both healthy and pain populations. However, when they only analyzed studies that assessed CPM using PPT as a test stimulus, they did find an association of state anxiety in healthy people. In a hypothetical model, fear or stressful emotions could activate inhibitory pathways, while depression and anxiety disorders could activate amygdala-induced facilitation processes [60]. In addition, anxiety and pain catastrophizing have been shown to be mediated by neurotransmitters such as serotonin and noradrenaline, which are also involved in the CPM response [61,62]. It is possible that an association with fear, catastrophizing and pain anxiety questionnaires may not be found because they assess general thoughts about pain at baseline instead of assessing the patient’s mental state during the painful stimulus. It is possible that in vivo scales such as the in vivo catastrophizing scale, which is assessed after experiencing pain, may better reflect these thoughts and may have an association with the inhibitory response. In the case of state anxiety, it is possible that the low scores obtained in participants are not sufficient to influence endogenous pain systems. No association between the immediate effects of PIMP and physical activity levels was found. In contrast, previous studies have demonstrated an association between higher levels of physical activity and a greater conditioned pain modulation (CPM) response in diverse populations, including healthy young individuals [63], healthy triathletes [64], and healthy older adults [65]. These findings were found when assessing the CPM response with heat as a test stimulus suggesting the influence of physical activity level on the hypoalgesic response to heat.

### 4.1. Clinical Implications

The present study contributes to the advancement of precision MT by proposing that its immediate effects may be mediated by CPM-related mechanisms. Clinicians can effectively apply these findings by inducing moderate pain through manual pressure, thereby eliciting immediate effects on pain sensitivity. It is important to note that repeating the stimulation at the same site or at different sites does not appear to enhance the effect. Nevertheless, it is crucial to exercise caution when applying these recommendations, as the study was conducted in pain-free participants, and the conclusions are contradictory in studies in the pain population. Future MT research in the pain population should be based on mechanism-based approaches that find subgroups of patients who may benefit from inhibitory effects related to moderate- or high-intensity pain-eliciting treatments. In addition, we should investigate what may be the optimal dosage when combined with exercise, which may also act through mechanisms related to CPM.

### 4.2. Strengths and Limitations

The current project exhibits several strengths that enhance its validity and reliability. Firstly, a noteworthy strength is the random assignment of participants to three distinct clinical trials. This strategic approach facilitated the analysis of multiple objectives within three highly comparable samples, enabling the connection of findings and leading to more robust and conclusive results. Secondly, the utilization of a crossover study design deserves recognition as it enables each participant to act as their own control, effectively minimizing the influence of between-subject variability. This design choice enhances the internal validity of the study. Lastly, the incorporation of a washout period of at least one day between interventions, coupled with the implementation of a mixed-effects analysis that incorporates day and sequence in the model, effectively addressed and controlled for potential risks associated with the crossover design. These actions helped to mitigate the possible influence of carryover effect, period effect, sequence effect, and period-by-treatment interaction on the results, further bolstering the reliability and validity of the study.

On the other hand, some limitations should be acknowledged. The main limitation of this study is the presence of a significant amount of missing data, particularly in Trial 1. These missing data reduce the power of the complete case analysis, which only considers participants who completed the entire sequence. To address this limitation, a mixed-effects model was employed as a valid alternative to incorporate all available data into the analysis. However, it is important to note that this approach assumes missingness is random or completely random. In the current project, it is plausible that the missing data were produced non-randomly, as some of the missing data were a result of exceptionally high PPT that exceeded the limits of the algometry device. This non-random missing data situation could potentially introduce bias and affect the generalizability of the results. Another limitation is that the sampling was carried out by convenience in three universities in the Autonomous Community of Madrid, obtaining a non-random sample of mostly young adult participants, which limits the external validity to the general population.

## 5. Conclusions

Manual pressure inducing moderate pain intensity produces a significant increase in PPT compared to pain-free manual pressure in healthy individuals. These findings suggest the involvement of CPM-related mechanisms. However, the effects on PPT induced through manual pressure may be significantly smaller than those induced by CPT, which are commonly used to assess CPM paradigm. The effects from PIMP were similar to those induced by electrostimulation. The intensity of the stimulus is a relevant factor, and a manual pressure that induces mild pain may not be sufficient for inducing effects on pain sensitivity. Conversely, increasing the dose of the manual pressure stimulus does not appear to have a significant impact on PPT, as the repetition of manual pressure stimuli does not increase the magnitude of the effect. Psychological factors and physical activity level do not appear to be associated with the inhibitory response observed in relation to PIMP.

## Figures and Tables

**Table 1:** Baseline characteristics of participants in the three trials.

		TRIAL 1 (*n* = 23)	TRIAL 2 (*n* = 24)	TRIAL 3 (*n* = 24)	Statistic	Between Groups *p*-Value
**Sex**	Female, No. (%)	13 (56.5%)	14 (58.3%)	8 (33.3%)	Chi^2^ = 3.71	0.156
	Male, No. (%)	10 (43.5%)	10 (41.7%)	16 (66.7%)		
**Age (y)**	Mean (SD)	24.8 (6.2)	23.6 (2.8)	23.9 (5.5)	H = 0.34	0.843
	Median (IQR)	23.3 (22.1, 25.5)	23.6 (21.4, 25.3)	23.4 (20.3, 25.3)		
**Height (m)**	Mean (SD)	1.7 (0.1)	1.7 (0.1)	1.7 (0.1)	F = 2.72	0.073
	Median (IQR)	1.7 (1.6, 1.8)	1.7 (1.6, 1.8)	1.7 (1.7, 1.8)		
**Weight (kg)**	Mean (SD)	64.1 (11.5)	66.1 (14)	67.7 (9.3)	F = 0.54	0.586
	Median (IQR)	62 (55, 70)	62.5 (56.5, 75.5)	68 (63, 72.8)		
**BMI (kg/m^2^)**	Mean (SD)	22.5 (3)	22.8 (2.9)	22.3 (2.2)	F = 0.23	0.793
	Median (IQR)	22.6 (19.9, 24.9)	22.7 (20.1, 24.8)	21.8 (20.6, 23.9)		
**PCS** **(0–52)**	Mean (SD)	5.7 (6.1)	7.7 (7.7)	6.8 (8.1)	H = 0.88	0.645
	Median (IQR)	3.5 (0, 11)	6 (1, 14)	4.5 (0, 10)		
**STAI-S** **(0–60)**	Mean (SD)	25.7 (3.3)	25.8 (4.3)	25.5 (4.2)	F = 0.03	0.968
	Median (IQR)	26.0 (22, 29)	26.5 (22.5, 29)	25.5 (22, 29)		
**PASS-20** **(0–100)**	Mean (SD)	17.7 (16.3)	26 (22.4)	20.6 (14.3)	H = 1.90	0.388
	Median (IQR)	12 (4, 33)	18.5 (8.5, 35)	16 (9.5, 32)		
**FPQ-III** **(0–45)**	Mean (SD)	17.9 (5.5)	20.5 (5.7)	18.8 (5.4)	F = 1.27	0.289
	Median (IQR)	19 (14, 21)	19.5 (16.5, 24)	19 (16, 22)		
**IPAQ (MET min/week)**	Mean (SD)	2743 (1380)	3706 (2881)	3340 (2095)	H = 0.50	0.779
	Median (IQR)	2671 (1838, 3680)	3439 (1386, 4080)	2853 (1515, 5484)		
**PPT (Kg/cm^2^)**	Mean (SD)	7.97 (2.28)	7.58 (2.51)	7.42 (2.28)	F = 0.32	0.724
	Median (IQR)	7.98 (6.04, 9.64)	7.32 (5.47, 9.08)	6.98 (6.15, 8.93)		

BMI = body mass index, FPQ-III = Fear of Pain Questionnaire-III, IPAQ = International Physical Activity Questionnaire, PASS-20 = Pain Anxiety Symptoms Scale, PCS = pain catastrophizing scale, PPT = pressure pain threshold, SD = standard deviation, STAI-S = State Anxiety Inventory, y = year.

**Table 2:** Effects of intervention, day, and sequence on changes in PPT in Trial 1.

Intervention	Pain-Inducing Manual Pressure (*n* = 20)	Pain-Inducing Electrical Stimulation (*n* = 21)	CPT (*n* = 22)	Difference between Interventions	
	*Mean (SD)*	*Mean (DS)*	*Mean (DS)*	*Chi^2^* *(p Value)*	*Differences (Bonferroni CI 95%)*
PPT pre-intervention	7.29 (2.61)	7.38 (2.13)	7.35 (2.09)	7.76 (0.021) *	Press vs. elec: −0.06 (0.82; 0.71) Press vs. cold: −0.77 (−1.53; −0.02) * Elec vs. cold: −0.72 (−1.46; 0.02)
PPT post-intervention	7.78 (2.68)	7.94 (2.46)	8.63 (2.66)		
Adjusted PPT post-intervention	*Marginals (CI 95%)*	*Marginals (CI 95%)*	*Marginals (CI 95%)*		
	7.86 (7.29; 8.42)	7.91 (7.36, 8.47)	8.63 (8.09; 9.17)		
**Day**	**Day 1 (*n* = 21)**	**Day 2 (*n* = 21)**	**Day 3 (*n* = 21)**	**Difference between days**	
	*Mean (SD)*	*Mean (DS)*	*Mean (DS)*	*Chi^2^* *(p value)*	*Differences (Bonferroni CI 95%)*
PPT pre-intervention	7.90 (2.31)	7.00 (2.26)	7.13 (2.15)	2.53 (0.283)	Day 1 vs. Day 2: −0.38 (−1.16; 0.38) Day 1 vs. Day 3: −0.48 (−1.26; 0.38) Day 2 vs. Day 3: −0.09 (−0.84; 0.65)
PPT post-intervention	8.41 (2.45)	7.86 (2.76)	8.11 (2.63)		
Adjusted PPT post-intervention	*Marginal (CI 95%)*	*Marginal (CI 95%)*	*Marginal (CI 95%)*		
	7.85 (7.29; 8.41)	8.24 (7.69; 8.80)	8.34 (7.79; 8.90)		
**Sequence**	**Seq 1 (*n* = 7)**	**Seq 2 (*n* = 8)**	**Seq 3 (*n* = 8)**	**Difference between sequences**	
	*Mean (SD)*	*Mean (DS)*	*Mean (DS)*	*Chi* ^2^ *(p value)*	*Differences (Bonferroni CI 95%)*
PPT pre-intervention	8.00 (2.21)	7.51 (2.32)	6.58 (2.06)	0.38 (0.827)	Seq 1 vs. Seq 2: −0.29 (−1.58; 1.00) Seq 1 vs. Seq 3: −0.29 (−1.58; 1.00) Seq 2 vs. 3 Seq: 0.00 (−1.26; 1.26)
PPT post-intervention	8.59 (2.57)	8.39 (2.73)	7.47 (2.44)		
Adjusted PPT post-intervention	*Marginal (CI 95%)*	*Marginal (CI 95%)*	*Marginal (CI 95%)*		
	7.95 (7.19; 8.71)	8.24 (7.50; 8.98)	8.24 (7.52; 8.96)		

* Significant differences (*p* < 0.05); CI = confidence intervals, CPT = cold pressor task, PPT = pressure pain threshold, SD = standard deviation, press = pressure, elec = electrical.

**Table 3:** Effects of intervention, day, and sequence on changes in PPT in Trial 2.

Intervention	Pressure 5/10 (*n* = 22)	Pressure 2/10 (*n* = 23)	Pressure 0/10 (*n* = 23)	Difference between Interventions	
	*Mean (SD)*	*Mean (DS)*	*Mean (DS)*	*Chi^2^* *(p Value)*	*Differences (Bonferroni CI 95%)*
PPT pre-intervention	7.61 (2.58)	7.38 (2.57)	7.33 (2.04)	9.72 (0.008) *	5/10 vs. 2/10: 0.79 (0.09; 1.48) * 5/10 vs. 0/10: 0.79 (0.09; 1.49) * 2/10 vs. 0/10: 0.01 (−0.68; 0.69)
PPT post-intervention	8.39 (2.22)	7.40 (2.39)	7.36 (2.13)		
Adjusted PPT post-intervention	*Marginal (CI 95%)*	*Marginal (CI 95%)*	*Marginal (CI 95%)*		
	8.24 (7.83; 8.65)	7.46 (7.06, 7.85)	7.45 (7.05; 7.85)		
**Day**	**Day 1 (*n* = 24)**	**Day 2 (*n* = 23)**	**Day 3 (*n* = 21)**	**Difference between days**	
	*Mean (SD)*	*Mean (DS)*	*Mean (DS)*	*Chi^2^* *(p value)*	*Differences (Bonferroni CI 95%)*
PPT pre-intervention	7.58 (2.51)	7.24 (2.43)	7.49 (2.25)	2.02 (0.364)	Day 1 vs. Day 2: 0.35 (−0.33; 1.04) Day 1 vs. Day 3: −0.00 (−0.70; 0.69) Day 2 vs. Day 3: −0.36 (−1.06; 0.35)
PPT post-intervention	7.96 (2.49)	7.29 (2.14)	7.88 (2.18)		
Adjusted PPT post-intervention	*Marginal (CI 95%)*	*Marginal (CI 95%)*	*Marginal (CI 95%)*		
	7.83 (7.44; 8.22)	7.47 (7.07; 7.87)	7.83 (7.41; 8.24)		
**Sequence**	**Seq 1 (*n* = 8)**	**Seq 2 (*n* = 8)**	**Seq 3 (*n* = 8)**	**Difference between sequences**	
	*Mean (SD)*	*Mean (DS)*	*Mean (DS)*	*Chi^2^* *(p value)*	*Differences (Bonferroni CI 95%)*
PPT pre-intervention	7.35 (2.75)	7.26 (2.50)	7.71 (1.83)	1.10 (0.577)	Seq 1 vs. Seq 2: −0.29 (−0.98; 0.39) Seq 1 vs. Seq 3: −0.20 (−0.90; 0.48) Seq 2 vs. Seq 3: 0.08 (−0.61; 0.78)
PPT post-intervention	7.48 (2.54)	7.67 (2.42)	7.98 (1.83)		
Adjusted PPT post-intervention	*Marginal (CI 95%)*	*Marginal (CI 95%)*	*Marginal (CI 95%)*		
	7.54 (7.14; 7.94)	7.83 (7.44; 8.23)	7.75 (7.34; 8.16)		

* Significant differences (p < 0.05); CI = confidence intervals, PPT = pressure pain threshold, SD = standard deviation.

**Table 4:** Effects of intervention, day, and sequence on changes in PPT in Trial 3.

Intervention	1. A Manual Pressure Stimulus (*n* = 22)	2. Repeated Manual Pressure (Same Point) (*n* = 23)	3. Repeated Manual Pressure (Different Points) (*n* = 22)	Difference between Interventions	
	*Mean (SD)*	*Mean (DS)*	*Mean (DS)*	*Chi^2^* *(p Value)*	*Differences (Bonferroni CI 95%)*
PPT pre-intervention	7.14 (2.08)	7.34 (2.03)	7.00 (1.94)	5.51 (0.064)	1 vs. 2: −0.45 (−1.19; 0.28) 1 vs. 3: −0.72 (−1.46; 0.02) 2 vs. 3: −0.27 (−1.01; 0.47)
PPT post-intervention	7.41 (1.99)	8.06 (2.21)	8.00 (2.60)		
Adjusted PPT post-intervention	*Marginal (CI 95%)*	*Marginal (CI 95%)*	*Marginal (CI 95%)*		
	7.44 (7.01; 7.87)	7.89 (7.47, 8.31)	8.16 (7.73; 8.59)		
**Day**	**Day 1 (*n* = 23)**	**Day 2 (*n* = 22)**	**Day 3 (*n* = 22)**	**Difference between days**	
	*Mean (SD)*	*Mean (DS)*	*Mean (DS)*	*Chi^2^* *(p value)*	*Differences (Bonferroni CI 95%)*
PPT pre-intervention	7.24 (2.14)	6.88 (1.78)	7.36 (2.09)	0.48 (0.787)	Day 1 vs. Day 2: 0.20 (−0.54; 0.94) Day 1 vs. Day 3: 0.03 (−0.71; 0.76) Day 2 vs. Day 3: −0.17 (−0.92; 0.58)
PPT post-intervention	7.96 (2.15)	7.43 (2.05)	8.09 (2.60)		
Adjusted PPT post-intervention	*Marginal (CI 95%)*	*Marginal (CI 95%)*	*Marginal (CI 95%)*		
	7.90 (7.48; 8.32)	7.70 (7.27; 8.14)	7.88 (7.44; 8.31)		
**Sequence**	**Seq 1 (*n* = 8)**	**Seq 2 (*n* = 8)**	**Seq 3 (*n* = 8)**	**Difference between sequences**	
	*Mean (SD)*	*Mean (DS)*	*Mean (DS)*	*Chi^2^* *(p value)*	*Differences (Bonferroni CI 95%)*
PPT pre-intervention	8.01 (1.81)	7.37 (2.07)	5.97 (1.55)	0.97 (0.616)	Seq 1 vs. Seq 2: 0.25 (−0.48; 0.98) Seq 1 vs. Seq 3: 0.29 (−0.51; 1.11) Seq 2 vs. Seq 3: 0.05 (−0.84; 0.74)
PPT post-intervention	8.84 (1.92)	7.96 (2.51)	6.53 (1.74)		
Adjusted PPT post-intervention	*Marginals (CI 95%)*	*Marginals (CI 95%)*	*Marginals (CI 95%)*		
	8.00 (7.58; 8.43)	7.75 (7.32; 8.18)	7.71 (7.24; 8.18)		

CI = confidence intervals, PPT = pressure pain threshold, SD = standard deviation.

**Table 5:** Multiple linear regressions assessing the association between the PIMP effect on PPT and anxiety status, pain anxiety, pain catastrophizing, fear of pain and physical activity.

Variables	*n*	Beta	SE	*p*	95% CI
**Anxiety state**	64	−0.01	0.35	0.754	−0.08, 0.06
PPT pre-intervention		0.86	0.06	0.000 *	0.74, 0.98
Sex		0.18	0.29	0.538	−0.40, 0.76
Age		0.02	0.03	0.500	−0.04, 0.08
**Pain anxiety**	64	−0.01	0.01	0.568	−0.02, 0.01
PPT pre-intervention		0.87	0.06	0.000 *	−0.04, 0.07
Sex		0.25	0.32	0.435	−0.39, 0.90
Age		0.01	0.03	0.609	−0.04, 0.72
**Pain Catastrophizing**	64	−0.02	0.02	0.385	−0.06, 0.02
PPT pre-intervention		0.86	0.06	0.000 *	0.75, 0.98
Sex		0.21	0.29	0.467	−0.37,0.79
Age		0.01	0.03	0.711	−0.05, 0.07
**Fear of pain**	64	0.03	0.03	0.310	−0.3, 0.08
PPT pre-intervention		0.85	0.06	0.000 *	0.73, 0.97
Sex		0.10	0.29	0.736	−0.48, 0.68
Age		0.03	0.03	0.362	−0.03, 0.08
**Physical activity**	56	0.00	0.00	0.553	−0.00, 0.00
PPT pre-intervention		0.88	0.06	0.000 *	0.75, 1.00
Sex		0.33	0.32	0.309	−0.31, 0.96
Age		0.02	0.03	0.534	−0.04, 0.08

* Significant differences (*p* < 0.05); CI = confidence intervals, PPT = pressure pain threshold, SE = standard error.

## Data Availability

The data presented in this study are available on request from the corresponding author. The data are not publicly available because the privacy of the research participants could be compromised.

## Supplementary Materials

The following supporting information can be downloaded at https://www.mdpi.com/article/10.3390/jcm13133648/s1, Table S1: Sensitivity analysis of trial 1 considering only subjects with no missing data; Table S2: Sensitivity analysis of trial 2 considering only subjects with no missing data; Table S3: Table S3. Sensitivity analysis of trial 3 considering only subjects with no missing data; Table S4. Assessment of washout at 30–60 min for each intervention; Table S5. Assessment of washout between days; Figure S1. PPT before and after each of the interventions in trial 1 (considering only subjects with no missing data); Figure S2. PPT before and after each of the interventions in trial 2 (considering only subjects with no missing data); Figure S3. PPT before and after each of the interventions in trial 3 (considering only subjects with no missing data).
