# Supplementary material for: CPM-Related Mechanisms Could Play a Key Role in the Effects on Pain Sensitivity Induced by Manual Therapy: Three Crossover Trials Investigating the Effects of Manual Pressure
Source: J Clin Med. 2024 Jun 22;13(13):3648. doi: 10.3390/jcm13133648 (PMC11242484; doi:10.3390/jcm13133648)
Supplement: Supplementary file 1 [file jcm-13-03648-s001.zip › jcm-3013878-supplementary.pdf]

**SUPPLEMENTARY MATERIAL**

**Table S1.** Sensitivity analysis of trial 1 considering only subjects with no missing data

| **Intervention** | **Pain-inducing manual pressure (n = 19)** | **Pain-inducing electrical stimulation (n = 19)** | **CPT (n = 19)** | **Difference between interventions** | |
|---|---|---|---|---|---|
| | *Mean (SD)* | *Mean (DS)* | *Mean (DS)* | *$Chi^2$ (p valor)* | *Differences (Bonferroni CI 95%)* |
| PPT pre-intervention | 7.39 (2.64) | 7.41 (2.20) | 7.31 (2.03) | 5.92 (0.052) | Pressure vs electrical: -0.02 (-0.83; 0.79)<br>Pressure vs cold: -0.72 (-1.53; 0.09)<br>Electrical vs cold: -0.70 (-1.52; 0.11) |
| PPT post-intervention | 7.85 (2.74) | 7.87 (2.47) | 8.50 (2.62) | | |
| | *Marginal (CI 95%)* | *Marginal (CI 95%)* | *Marginal (CI 95%)* | | |
| Adjusted PPT post-intervention | 7.83 (7.22; 8.43) | 7.84 (7.24, 8.44) | 8.55 (7.95; 9.15) | | |
| **Day** | **Day 1 (n = 19)** | **Day 2 (n = 19)** | **Day 3 (n = 19)** | **Difference between days** | |
| | Mean (SD) | Mean (DS) | Mean (DS) | *$Chi^2$ (p valor)* | Differences (Bonferroni CI 95%) |
| PPT pre-intervention | 7.91 (2.23) | 7.15 (2.33) | 7.06 (2.25) | 1.94 (0.379) | Day 1 vs Day 2: -0.40 (-1.23; 0.42)<br>Day 1 vs Day 3: -0.44 (-1.27; 0.40)<br>Day 2 vs Day 3: -0.03 (-0.85; 0.78) |
| PPT post-intervention | 8.28 (2.30) | 8.01 (2.86) | 7.93 (2.69) | | |
| | *Marginal (CI 95%)* | *Marginal (CI 95%)* | *Marginal (CI 95%)* | | |
| Adjusted PPT post-intervention | 7.79 (7.18; 8.40) | 8.20 (7.59; 8.80) | 8.23 (7.63; 8.84) | | |
| **Sequence** | **Seq 1 (n = 6)** | **Seq 2 (n = 7)** | **Seq 3 (n = 6)** | **Difference between sequences** | |
| | *Mean (SD)* | *Mean (DS)* | *Mean (DS)* | *$Chi^2$ (p valor)* | *Differences (Bonferroni CI 95%)* |
| PPT pre-intervention | 8.17 (2.26) | 7.51 (2.32) | 6.41 (1.94) | 0.35 (0.839) | Seq 1 vs Seq 2: -0.31 (-1.69; 1.08)<br>Seq 1 vs Seq 3: -0.03 (-1.51; 1.45)<br>Seq 2 vs Seq 3: 0.27 (-1.12; 1.67) |
| PPT post-intervention | 8.69 (2.66) | 8.39 (2.73) | 7.09 (2.14) | | |
| | *Marginal (CI 95%)* | *Marginal (CI 95%)* | *Marginal (CI 95%)* | | |
| Adjusted PPT post-intervention | 7.95 (7.11; 8.79) | 8.26 (7.49; 9.02) | 7.98 (7.14; 8.83) | | |

* Significant differences ($p < 0.05$); *CI* = confidence intervals, *CPT* = cold pressor task, *PPT* = pressure pain threshold, SD = standard deviation

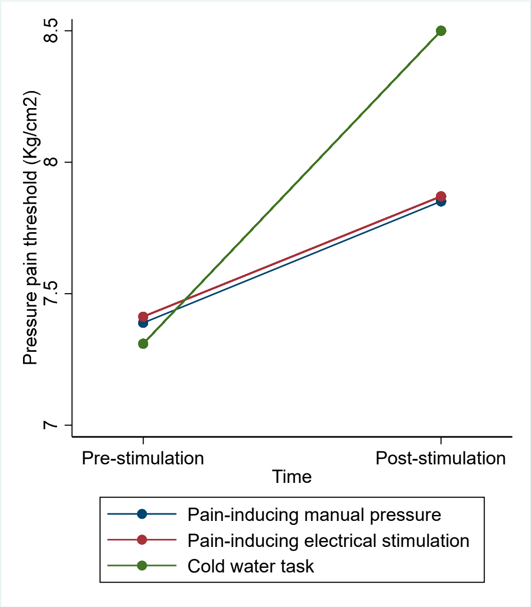


**Figure S1**. PPT before and after each of the interventions in trial 1 (considering only subjects with no missing data)

**Table S2.** Sensitivity analysis of trial 2 considering only subjects with no missing data

| **Intervention** | **Pressure 5/10 (n = 21)** | **Pressure 2/10 (n = 21)** | **Pressure 0/10 (n = 21)** | **Difference between interventions** | |
|---|---|---|---|---|---|
| | *Mean (SD)* | *Mean (DS)* | *Mean (DS)* | *Chi² (p valor)* | *Differences (Bonferroni CI 95%)* |
| PPT pre-intervention | 7.45 (2.53) | 7.36 (2.36) | 7.43 (1.96) | 8.49 (0.014)* | 5/10 vs 2/10: 0.76 (0.06; 1.45)*<br>5/10 vs 0/10: 0.71 (0.011; 1.40)*<br>2/10 vs 0/10: -0.05 (-0.75; 0.65) |
| PPT post-intervention | 8.21 (2.10) | 7.37 (2.05) | 7.48 (2.05) | | |
| Adjusted PPT post-intervention | *Marginal (CI 95%)*<br>8.18 (7.77; 8.58) | *Marginal (CI 95%)*<br>7.42 (7.02, 7.82) | *Marginal (CI 95%)*<br>7.47 (7.07; 7.87) | | |
| **Day** | **Day 1 (n = 21)** | **Day 2 (n = 21)** | **Day 3 (n = 21)** | **Difference between days** | |
| | Mean (SD) | Mean (DS) | Mean (DS) | *Chi² (p valor)* | Differences (Bonferroni CI 95%) |
| PPT pre-intervention | 7.56 (2.40) | 7.20 (2.22) | 7.49 (2.25) | 2.88 (0.236) | Day 1 vs Day 2: 0.44 (-0.26; 1.14)<br>Day 1 vs Day 3: 0.02 (-0.68; 0.71)<br>Day 2 vs Day 3: -0.41 (-1.12; 0.28) |
| PPT post-intervention | 7.95 (2.23) | 7.23 (1.77) | 7.88 (2.18) | | |
| Adjusted PPT post-intervention | *Marginals (CI 95%)*<br>7.83 (7.44; 8.24) | *Marginals (CI 95%)*<br>7.40 (7.00; 7.81) | *Marginals (CI 95%)*<br>7.82 (7.42; 8.23) | | |
| **Sequence** | **Seq 1 (n = 7)** | **Seq 2 (n = 7)** | **Seq 3 (n = 7)** | **Difference between sequences** | |
| | *Mean (SD)* | *Mean (DS)* | *Mean (DS)* | *Chi² (p valor)* | *Differences (Bonferroni CI 95%)* |
| PPT pre-intervention | 6.98 (2.57) | 7.61 (2.33) | 7.66 (1.86) | 3.76 (0.152) | Seq 1 vs Seq 2: -0.56 (-1.25; 0.14)<br>Seq 1 vs Seq 3: -0.38 (-1.08; 0.32)<br>Seq 2 vs Seq 3: 0.17 (-0.521; 0.87) |
| PPT post-intervention | 7.03 (2.15) | 8.08 (2.10) | 7.95 (1.87) | | |
| Adjusted PPT post-intervention | *Marginals (CI 95%)*<br>7.38 (6.97; 7.78) | *Marginals (CI 95%)*<br>7.93 (7.53; 8.34) | *Marginals (CI 95%)*<br>7.76 (7.35; 8.16) | | |

** Significant differences ($p < 0.05$); CI = confidence intervals, PPT = pressure pain threshold, SD = standard deviation*

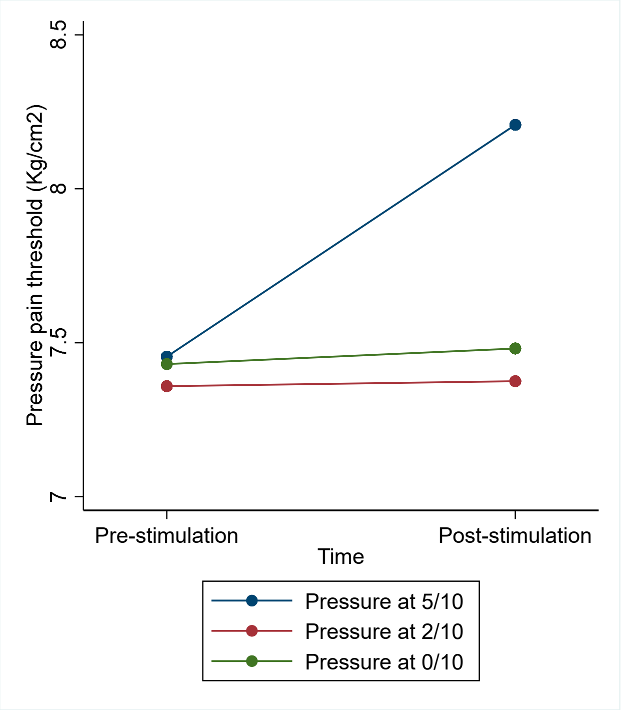


**Figure S2**. PPT before and after each of the interventions in trial 2 (considering only subjects with no missing data)

**Table S3.** Sensitivity analysis of trial 3 considering only subjects with no missing data

| **Intervention** | **1. A manual pressure stimulus (n = 22)** | **2. Repeated manual pressure (same point) (n = 22)** | **3. Repeated manual pressure (different points) (n = 22)** | **Difference between interventions** | |
|---|---|---|---|---|---|
| | *Mean (SD)* | *Mean (DS)* | *Mean (DS)* | *Chi$^2$ (p valor)* | *Differences (Bonferroni CI 95%)* |
| PPT pre-intervention | 7.14 (2.08) | 7.11 (1.73) | 7.00 (1.94) | 5.46 (0.065) | 1 vs 2: -0.39 (-1.13; 0.34)<br>1 vs 3: -0.71 (-1.44; 0.02)<br>2 vs 3: -0.32 (-1.05; 0.41) |
| PPT post-intervention | 7.41 (1.99) | 7.78 (1.78) | 8.00 (2.60) | | |
| Adjusted PPT post-intervention | *Marginal (CI 95%)*<br>7.36 (6.93; 7.79) | *Marginal (CI 95%)*<br>7.76 (7.33, 8.18) | *Marginal (CI 95%)*<br>8.07 (7.64; 8.50) | | |
| **Day** | **Day 1 (n = 22)** | **Day 2 (n = 22)** | **Day 3 (n = 22)** | **Difference between days** | |
| | Mean (SD) | Mean (DS) | Mean (DS) | Chi$^2$ (p valor) | Differences (Bonferroni CI 95%) |
| PPT pre-intervention | 7.00 (1.85) | 6.88 (1.78) | 7.36 (2.09) | 0.42 (0.812) | Day 1 vs Day 2: 0.15 (-0.58; 0.88)<br>Day 1 vs Day 3: -0.04 (-0.78; 0.69)<br>Day 2 vs Day 3: -0.19 (-0.93; 0.55) |
| PPT post-intervention | 7.67 (1.69) | 7.43 (2.05) | 8.09 (2.60) | | |
| Adjusted PPT post-intervention | *Marginal (CI 95%)*<br>7.77 (7.34; 8.19) | *Marginal (CI 95%)*<br>7.62 (7.19; 8.05) | *Marginal (CI 95%)*<br>7.81 (7.38; 8.24) | | |
| **Sequence** | **Seq 1 (n = 8)** | **Seq 2 (n = 7)** | **Seq 3 (n = 7)** | **Difference between sequences** | |
| | *Mean (SD)* | *Mean (DS)* | *Mean (DS)* | *Chi$^2$ (p valor)* | *Differences (Bonferroni CI 95%)* |
| PPT pre-intervention | 8.01 (1.81) | 7.13 (1.77) | 5.97 (1.55) | 1.53 (0.465) | Seq 1 vs Seq 2: 0.34 (-0.43; 1.11)<br>Seq 1 vs Seq 3: 0.38 (-0.46; 1.21)<br>Seq 2 vs Seq 3: 0.04 (-0.76; 0.84) |
| PPT post-intervention | 8.84 (1.92) | 7.66 (2.12) | 6.53 (1.74) | | |
| Adjusted PPT post-intervention | *Marginal (CI 95%)*<br>7.96 (7.52; 8.40) | *Marginal (CI 95%)*<br>7.62 (7.17; 8.07) | *Marginal (CI 95%)*<br>7.58 (7.10; 8.06) | | |

* Significant differences ($p < 0.05$); *CI* = confidence intervals, *PPT* = pressure pain threshold, SD = standard deviation

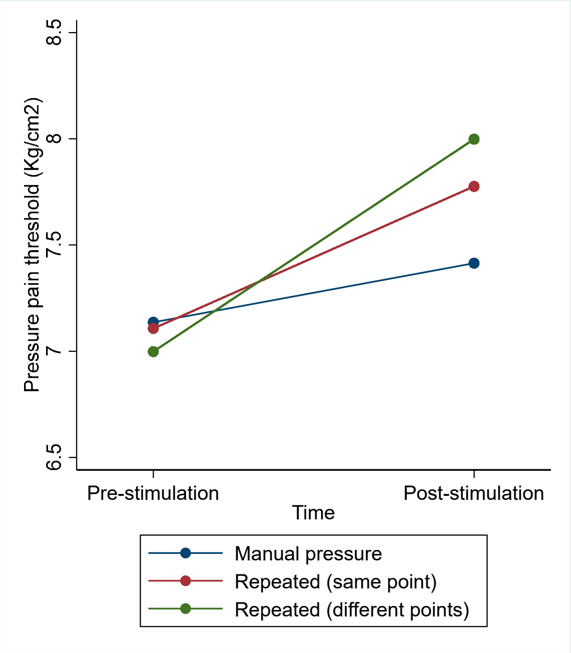


**Figure S3**. PPT before and after each of the interventions in trial 3 (considering only subjects with no missing data)

**Table S4.** Assessment of washout at 30-60 minutes for each intervention.

| Study | Intervention | PPT pre-intervention | PPT post-30-60 min | Difference | | |
|---|---|---|---|---|---|---|
| | | Mean (SD) | Mean (SD) | Mean (CI 99%) | Statistic t | P value |
| Trial 1 | Pain-inducing manual pressure (n=19) | 7.17 (2.45) | 7.47 (2.18) | -0.30 (1.23; 0.62) | -0.94 | 0.358 |
| | Pain-inducing electrical stimulation (n=19) | 7.40 (2.19) | 7.69 (2.25) | -0.29 (-1.06; 0.44) | -1.13 | 0.273 |
| | Cold water task (n=19) | 7.14 (2.03) | 7.54 (2.52) | -0.40 (-1.05; 0.25) | -1.77 | 0.093 |
| Trial 2 | Pressure 5/10 (n=20) | 7.45 (2.63) | 7.32 (1.74) | 0.13 (-0.92; 1.18) | 0.35 | 0.730 |
| | Pressure 2/10 (n=23) | 7.38 (2.57) | 7.54 (2.61) | -0.16 (-0.74; 0.42) | -0.77 | 0.45 |
| | Pressure 0/10 (n=22) | 7.40 (2.06) | 7.05 (1.97) | 0.35 (-0.11; 0.80) | 2.16 | 0.042 |
| Trial 3 | Manual pressure (n=21) | 7.51 (2.56) | 7.57 (2.58) | -0.06 (-0.53; 0.42) | -0.35 | 0.730 |
| | Repeated manual pressure (same point) (n=23) | 7.34 (2.03) | 7.52 (1.95) | -0.17 (-0.62; 0.27) | 1.09 | 0.287 |
| | Repeated manual pressure (different points) (n=21) | 6.96 (1.98) | 7.26 (2.44) | -0.30 ( -1.03; 0.44) | -1.14 | 0.266 |

* Significant differences ($p < 0.01$); *CI* = confidence intervals, *PPT* = pressure pain threshold, SD = standard deviation

**Table S5.** Assessment of washout between days

| Study | | Day 1 | Day 2 | Day 3 | Difference | | |
|---|---|---|---|---|---|---|---|
| | | Mean (SD) | Mean (SD) | Mean (SD) | Statistic F | P value | Contrasts (Bonferroni CI 95%) |
| Trial 1 (n = 21) | PPT pre-intervention | 7.84 (2.25) | 7.00 (2.26) | 6.96 (2.18) | 1.04 | 0.360 | 2 vs 1: -0.84 (-2.54, 0.85)<br>3 vs 1: -0.88 (-2.57, 0.82)<br>3 vs 2: -0.03 (-1.73, 1.66) |
| Trial 2 (n = 21) | PPT pre-intervention | 7.56 (2.40) | 7.20 (2.22) | 7.48 (2.24) | 0.15 | 0.863 | 2 vs 1: -0.36 (-2.11, 1.38)<br>3 vs 1: -0.08 (-1.82, 1.66)<br>3 vs 2: 0.29 (-1.45, 2.03) |
| Trial 3 (n = 22) | PPT pre-intervention | 7.00 (1.85) | 6.88 (1.78) | 7.36 (2.09) | 0.39 | 0.681 | 2 vs 1: -0.12 (-1.54, 1.29)<br>3 vs 1: 0.36 (-1.05, 1.78)<br>3 vs 2: 0.49 (-0.93, 1.91) |

* Significant differences ($p < 0.05$); *CI* = confidence intervals, *PPT* = pressure pain threshold, SD = standard deviation
